# Planning of graft size and 3D reconstruction using virtual reality technique in aortic valve reimplantation

**DOI:** 10.3389/fcvm.2022.1064617

**Published:** 2023-01-11

**Authors:** Gregory Reid, Julian Gehweiler, Florian Thieringer, Friedrich Eckstein, Enrico Ferrari, Brigitta Gahl, Denis A. Berdajs

**Affiliations:** ^1^Department of Cardiac Surgery, University Hospital Basel, Basel, Switzerland; ^2^Department of Radiology, University Hospital Basel, University of Basel, Basel, Switzerland; ^3^Medical Additive Manufacturing Research Group, Department of Biomedical Engineering, University of Basel, Allschwil, Switzerland; ^4^Cardiac Surgery, Istituto Cardiocentro Ticino, Lugano, Switzerland

**Keywords:** aortic root, geometry of the aortic root, aortic valve reimplantation, 3D modeling, virtual surgical planning

## Abstract

**Objectives:**

To evaluate applicability and feasibility of the virtual imaging technology for diagnosis and planning of the aortic valve sparing procedure.

**Methods:**

Pre-operative electrocardiography-gated computed tomography images of 12 adult patients with aortic root pathology were used for 3D reconstruction of the aortic root geometry. The structural analysis was conducted with focusing on spatial architecture of key aortic root structures such as the three commissures, intervalvular triangles (IVT), as well as on morphology of the aortic root base (AoB) and of the sinotubular junction (STJ).

**Results:**

In all included patients, the 3D mapping of aortic root (AoR) morphology was successfully performed. The pre-operative diameter of the AoB was 30.6 ± 2.6 mm and of the STJ 46.5 ± 7.5 mm (*p* < 0.001). Based on measured AoB diameter, the mean size of prosthesis used was 28.3 ± 1.37 mm. The planar arrangement of the three commissures was similar to an equilateral triangle where the three commissures were at similar distance for each individual sinus with 39.8 ± 6.64 mm for right, 37.5 ± 7.10 mm for left, and 39.2 ± 7.52 mm for non-coronary sinus (*p* = 0.72) subsequently. The similar height of the three IVT’s with 32.6 ± 5.87 mm for right, 33.6 ± 6.14 mm for anterior, and 31.7 ± 5.83 mm for left IVT (*p* = 0.73) was suggestive for all three commissures being positioned in the same plane. Consequently at reimplantation, the orientation of the three commissures followed the pattern of an equilateral triangle.

**Conclusion:**

The reconstructed images revealed a detailed 3D anatomy of the aortic root, with the spatial arrangement of the intervalvular triangles, planimetric orientation of the commissures, as well as determination of the AoB and STJ diameters. Obtained information was successfully applied to pre-operative surgical planning. The reimplantation technique, the height of the reimplanted intervalvular triangles, as well as their orientation are crucial for achieving adequate aortic valve function.

## 1. Introduction

In the past 10 years, aortic root (AoR) reconstruction has become increasingly popular in the treatment of AoR dilatation. Reconstructive procedures such as David (reimplantation), Yacoub (remodeling) and recently introduced AoR sparing with annular ring implantation (CAVIAAR) all have the common intention to avoid the disadvantages of the mechanical or biological prosthesis (1). Despite the excellent long term outcome in experienced centers and net effort for standardization of the valve sparing procedure, a graft size selection method has not yet been established and remains controversial (2, 3), probably because aortic valve reimplantation is a technically demanding and complex procedure (4). Furthermore, in most reports with long term outcome the technique is downgraded to the aortic valve replacement with limited information on AoR pathology and its surgical management. In recent literature impression is given that aortic root reimplantation is a reproducible and standardized surgical procedure (4–6).

Fact is, that in dilatative pathology, the aortic root dysfunction is closely linked to the morphological alteration. Intuitively, one can suggest that the detailed perception of the 3D AoR architecture may play a key role in successful restoration of the AoR as a functional unit. Indeed, in the field of structural valve interventions it has been shown that patient specific 3D image models are useful tools for precise geometric observation, clinical education, and pre-procedural planning of interventional cases (7, 8).

Detailed 3D mapping of the aortic root pathological morphology as a tool for pre-operative planning for aortic root repair has not been described yet.

Adding such detailed pre-operative information on aortic root pathology may be an important step toward the standardization of the procedure, as well as the introduction of patient specific customization of the intervention. The aim of this study was to apply a virtual 3D reconstruction technology for the development of 3D AoR anatomy reconstruction as a tool for pre-operative diagnosis and planning.

## 2. Materials and methods

In 12 consecutive patients with aortic valve insufficiency, aortic valve reimplantation technique was performed for dilatative AoR pathology. Coronary angiography, electrocardiograph-gated multislice computed tomography scan (CT) and echocardiography were performed in all patients. The pre-operative echocardiography was used for evaluation of the aortic valve pathology and for quantification of the valve insufficiency. Prior to discharge echocardiography was performed to evaluate valve function.

### 2.1. Ethics statement

The study protocol, of this retrospective study, was approved by the local Ethical Committee at the University of Basel, Basel, Switzerland, (Ethikkomission Nordwest und Zentralschweiz, EKNZ 2021-02323). A written informed consent was waived due to the retrospective nature of the study.

### 2.2. 3D data acquisition

The 3D data acquisition involved several steps. Prior to segmentation, the acquired CT scan images were exported into a Digital Imaging and Communications in Medicine (DICOM). From the DICOM data set, the target AoR geometry was identified and segmented by Materialise mimics^©^ software (Materialise, Leuven, Beligum). The segmentation masks were converted into 3D digital models using rendering techniques. These patient-specific 3D digital models of AoR were saved as a stereolithography file and prepared for further measurements (9).

**FIGURE 1:**
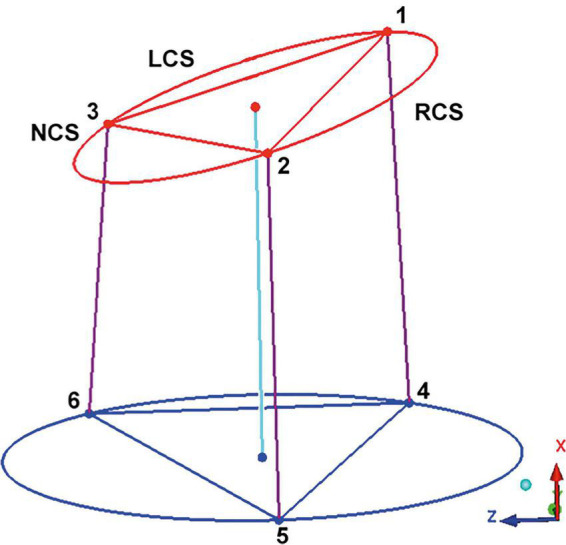
Geometric model of the aortic root. The “*123*” triangle is positioned in the sinotubular junction. The three edges correspond to the left (LCS), right (RCS), and non (NCS) coronary sinus commissures. The triangle at the aortic root base is marked by the “*456*” triangle, the basal points correspond to the vertical projection of the three commissures into the root base. By interconnecting the points of both triangles, a model of the aortic root is obtained. This model is a prism where the basal and the sinotubular junction plane are not parallel. The axe of the prism interconnect the center of the sinotubular junction and aortic root base. LCS, left coronary sinus; RCS, right coronary sinus; NCS, non-coronary sinus. Adapted from (16). Copyright Nr 5456880811785.

For assessment of the AoR 3D geometry, previously defined morphological landmarks were applied. Briefly, the cranial border of the AoR was defined by the sinotubular junction (STJ). At this level, the three commissures: the anterior, the left, and the right commissure together describe a triangle. The AoR base (AoB), which is a virtual structure, is seen as the deepest point of each individual sinus and of the base of intervalvular triangles (IVT). The mid-point of the each individual IVT was chosen as a landmark at the AoR base. The three basal landmarks at the anterior, left, and right IVT are counterpart elements of the three commissures and also define a triangle (10, 11; Figure 1).

**FIGURE 2:**
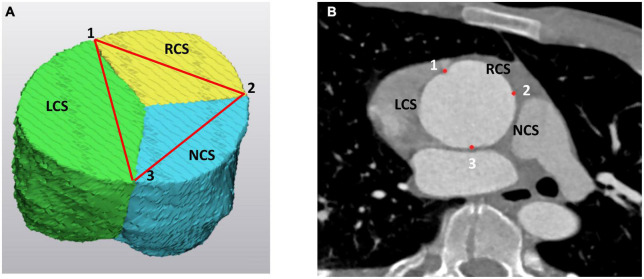
The three commissures at the level of the sinotubular junction (STJ) as **(A)** on the 3D model of the aortic root and **(B)** on the angio CT scan. The “*123*” landmarks correspond to the left (LCS), right (RCS), and non (NCS) coronary sinus commissures. On the 3D model the LCS is marked as green, the RCS is yellow and the NCS is blue. LCS, left coronary sinus; RCS, right coronary sinus; NCS, non-coronary sinus.

**FIGURE 3:**
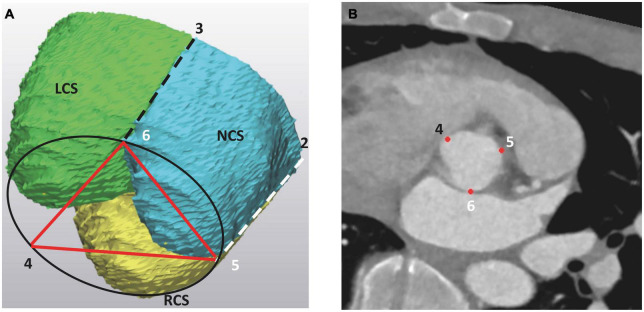
The basal landmarks of the aortic root at the level of the aortic root base (AoB), as seen **(A)** on the 3D model of the aortic root and **(B)** on the angio CT scan. The basal landmarks “*456*” correspond to the deepest points at each IVT and are vertical projections of the three commissures into the AoB. On the 3D model, the LCS is marked as green, the RCS is yellow and the NCS is blue. The projection of the left and right commissure to the aortic root base is indicated by dashed lines. LCS, left coronary sinus; RCS, right coronary sinus; NCS, non-coronary sinus.

**FIGURE 4:**
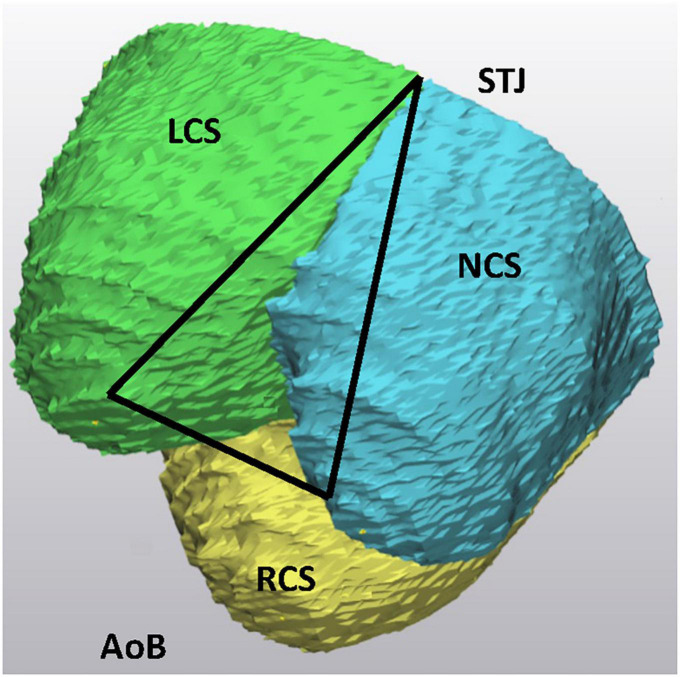
The intervalvular triangle, as seen on 3D models of the aortic root. The peak of the triangle corresponds to the commissure at the level of the STJ. AoB, aortic root base; STJ, sinotubular junction; LCS, left coronary sinus; RCS, right coronary sinus; NCS, non-coronary sinus.

After the patient specific 3D model of the AoR was obtained, the following distances were measured:

(a) the intercommissural distances at the STJ (Figure 2),

(b) basal distances between the three landmarks at the base of each IVT (Figure 3),

(c) the height of each IVT (Figure 4).

Area-derived diameters at the AoB, STJ and at the middle part of the Sinus Valsalva were measured centerline to the flow direction. The volume of each individual Sinus Valsalva was obtained. The length of the leaflet coaptation margin was measured along the leaflet margin from one commissure to another.

#### 2.2.1. Reproducibility of the 3D model

All geometric parameters including segmentation and creation of 3D model were consistently measured by one physician (R.G.). Variability was compared with blinded measurements of a radiologist with 10 years of research experience (J.G). For any deviation of more than 2 mm measurements were repeated and arbitrary distance was set.

### 2.3. Surgical approach; aortic valve reimplantation

In all included patients, aortic valve reimplantation was performed by using a Gelweave Valsalva graft (Vascutek Ltd., Inchinnan, United Kingdom). The surgical technique of the aortic valve reimplantation has been described in detail in the past (12–15). In short, following the institution of cardiopulmonary bypass and cardioplegic arrest, the aorta is transected 1 cm above the STJ. The retraction sutures are placed at each commissure, dissection of the aortic root is performed and the sinuses of Valsalva are resected. The coronary buttons are isolated and harvested (13–15).

#### 2.3.1. Implementation of 3D data in reimplantation technique

The graft diameter size is estimated based on the measured diameter at the AoB in the 3D model of the aortic root. The measured diameter provides *de facto* an internal diameter at the AoB since the graft is placed around the aortic valve. To accommodate this, the aortic root base is slightly undersized. As a double check for graft size, the aortic root base diameter is also measured by use of a mechanical valve sizer (St Jude Medical).

After the proximal suture line is completed using twelve 2-0 tycoon pledged sutures, the orientation and fixation of the three IVT’s respecting the placement of three commissures is conducted. The height the IVT’s measured in the 3D model determines the level of the reimplanted commissures in the graft. This height does not have to be the same, consequently the three commissures can be reimplanted at different levels. After the basal sutures are passed through the base of the graft, the three commissures are pulled in a vertical direction. The height of each IVT corresponds to the level of the reimplantation of each commissure.

The orientation of the three commissures follows the geometry of the intercommissural distances, measured in a 3D model, assuming that three commissures represent corners of a triangle.

#### 2.3.2. Aortic valve assessment

The aortic valve function was evaluated immediately post intervention by transesophageal color Doppler echocardiography, as well as at discharge by trans-thoracic color Doppler echocardiography. The aortic valve insufficiency (AI) was assessed with following criteria: 0 = none; I = minimal; II = mild; III = moderate; and IV = severe (15).

### 2.4. Statistical analyses

Quantitative parameters are expressed as mean ± standard deviation. We compared anatomic measurements among the three sinuses groups using paired *t*-tests and Wilcoxon matched-pairs signed-rank tests as a sensitivity analysis for each tuple of variables. Our statistical analyses were however, exploratory in nature. *p*-values less than 0.05 were considered statistically significant. Analyses have been conducted using Stata 16.0 (StataCorp, College Station, TX, USA).

## 3. Results

### 3.1. Baseline characteristics

Pre-operative patients’ characteristics are presented in Table 1, the mean age of patients included was 52.25 ± 10.64 and 3 (25%) were females. None of the patients has had history of renal impairment of history of myocardial infarction. The aortic valve was in all cases tricuspid. The left and the right ventricular functions were preserved and in *n* = 7 patients ≥ II grade aortic valve insufficiency was diagnosed. The peri-operative data are presented in Table 2. The mean hospital stay was 11.67 (7–26) days, there was no mortality registered during the hospital stay.

**TABLE 1:** Patient demographics and pre-operative echocardiographic data.

Patient characteristics	Patients (*N* = 12)
Age (Years)	52.25 ± 10.64
Gender (Female)	3 (25%)
Arterial hypertension	8 (66.7%)
Positive family history	2 (16.7%)
History of stroke	2 (16.7%)
Dyslipidemia	6 (50%)
EuroScore II	0.95 ± 0.2
Body surface area (BSA)	2.00 ± 0.24
**Aortic insufficiency (AI) (Preop):**	
AI 0	3 (25%)
AI I	2 (16.7%)
AI II	2 (16.7%)
AI > II	5 (42.7%)
Mean gradient (mmHg)	4.86 ± 2.59
LVEF (%)	57.4 ± 7.41
VC (mm)	3.25 ± 2.52
LVEDD (mm)	53.8 ± 8.13

AI, aortic valve insufficiency; BSA, body surface; LVEF, left ventricular ejection fraction; LVEDD, left ventricular end diastolic diameter; VC, vena contracta.

**TABLE 2:** Peri-operative outcome.

Intraoperative characteristics	Patients (*N* = 12)
Operation length (min)	269.25 ± 61.63
Cardiopulmonary bypass time (min)	189.42 ± 47.01
Aortic cross clamp time (min)	157.42 ± 37.93
Lowest core temperature (°C)	26.32 ± 4.68
**ICU stay:**	
<24 h	8 (66.7%)
24–48 h	3 (25%)
48–72 h	1 (8.3%)
Hospital stay (d)	11.67 (7–26)

ICU, intensive care unit.

### 3.2. 3D assessment of the aortic root

The 3D reconstruction of the aortic root morphology was successfully conducted in all cases. The mean surface derivate diameter of the AoB was 30.05 ± 2.61 mm, the STJ was 46.56 ± 7.49 mm and of the Sinus Valsalva measured at the mid distance between the AoB and STJ was 49.2 ± 6.06 mm. The mean size of the aortic root annulus measured with the mechanical prosthesis sizer was 27.8 ± 2.37 mm (St Jude Medical).

The diameter ratio between AoB and STJ was 66.69 ± 14.66%. The average volumes of the non-coronary (NCS) and the right coronary sinus (RCS) were similar at 18.94 ± 7.4 ml and 18.14 ± 6.35 ml, respectively, and slightly larger than the average volume measured in the left coronary sinus (LCS) at 16.23 ± 5.98 ml (*p* = 0.59).

The distances between the commissures at the STJ were as follows: 39 ± 6.64 mm for the RCS, 37.0 ± 7.11 mm for the LCS and 39.18 ± 7.54 mm (*p* = 0.71) for the NCS. The corresponding length at the AoB, measuring the distance between the intervalvular triangles were: in the RCS 24.85 ± 2.95 mm, in the LCS 24.18 ± 3.15 mm and in the NCS 26.61 ± 3.91 mm (*p* = 0.20). The height of each individual intervalvular triangle was not significantly different: right IVT 32.61 ± 5.87 mm, anterior IVT 33.64 ± 6.14 mm and the left IVT was 31.74 ± 5.83 mm, (*p* = 0.72). The coaptation length at the end of the diastole measured 24.46 ± 11.73 mm for the non-coronary leaflet, 23.49 ± 11.18 mm for the right leaflet and 24.74 ± 11.97 mm (*p* = 0.99) for the left leaflet of the aortic valve.

The mean size of the aortic root prosthesis graft (Gelweave Valsalva graft Vascutek Ltd., Inchinnan, United Kingdom) chosen for reimplantation is based on measurements of the AoB size in the 3D CT model in all patients and was 28.8 ± 1.24 mm. Since the three intervalvular triangles were shown in the pre-operative measurement to have almost similar heights, the three posts were reimplanted at the same level. In this way a neo STJ was almost parallel to the AoB. The orientation of the three commissures corresponded to the geometry of an equilateral triangle, consequently the three commissures were reimplanted in similar distances from each other. The orientation corresponded to the three symmetrical markings of the sinus Valsava prosthesis.

### 3.3. Echocardiographic findings

The pre-operative and post-operative echocardiographic data are presented in Table 3. In early post-interventional obtained transthoracic echocardiogram, the aortic valve without insufficiency was registered in eight patients and AI grade I was present in four patients with a vena contracta of 1.12 ± 0.12 mm. All four patients of these patients had AI grade III before intervention.

**TABLE 3:** Grading of the aortic valve insufficiency after the reimplantation of the aortic valve.

Aortic insufficiency (AI) (Postop)	Patients (*N* = 12)
AI 0	8 (66.67%)
AI I	4 (33.33%)
AI II	0
AI > II	0
VC (mm)	0.39 ± 1.56
LVEF (%)	55.4 ± 5.71
Mean gradient (mmHg)	5.82 ± 3.75

AI, aortic valve insufficiency; LVEF, left ventricular ejection fraction; VC, vena contracta.

## 4. Discussion

In the present report we successfully applied virtual 3D reconstruction as an image editing tool for a representation of the aortic root reimplantation. In the 3D models, the morphology of each individual aortic root was assessed focusing on the geometrical arrangement of the aortic root landmarks playing a crucial role in proper aortic valve function: on the position of the three IVT’s, on the arrangement of the three commissures at the level of the STJ and their projection to the aortic root base. The spatial arrangement of the landmarks follows a strict 3D architecture and was in the past defined as a prism (10, 11).

The IVT’s are considered as a skeleton of the AoR, serving as an anchoring pillar for the attachment of the three leaflets. In normal AoR anatomy, the IVT’s are positioned in a vertical direction and the orientation is crucial for correct aortic valve function. Any deviation from the vertical direction of the IVT’s, as for example in dilatation of the STJ, results in varying degrees of aortic leaflets coaptation and consequently may lead to an incompetent function. In the 3D reconstruction models of AoR CT scans, deviations from the vertical direction of the IVT’s may be visualized, however, the grade of deviation from the vertical direction is not assessed. We assumed that numerical description of IVT’s deviation form vertical direction wouldn’t have an important impact on planning of the surgical intervention. One must consider that verticalization of the IVT’s is one of the important steps in the reimplantation procedure providing maximal coaptation surface of the aortic valve (2, 13–15).

In analysis of the three commissures in the 3D models, we in first line were evaluating the planar constellation of the three commissures. From a geometrical point of view, the three commissures may be considered as the edges of a triangle inscribed in a circle (Figure 2). Ideally this triangle may be considered as an equilateral triangle where all three distances between the commissures are the same or similar. It is self-explanatory that the orientation of the reimplanted commissures should follow their natural planar arrangement. Deviation from the given inter-commissural arrangement in the reimplanted root may lead to incompetent valve coaptation, with leaflet restriction on the side of the distance augmentation and to the leaflet prolapse at the side of distance reduction. Indeed, in our cohort, average values of the inter-commissural distances were not significantly different and one may assume that in all 12 cases investigated, the commissures were positioned in the graft corresponding to the orientation of the equilateral triangle. In four patients, the inter-commissural distance of the RCS was larger than those assessed in the LCS and NCS. Consequently, the commissures were reimplanted respecting the natural asymmetry to obtain an optimal leaflet coaptation. At discharge, out of these four patients, three had no evidence of residual aortic valve insufficiency and one was discharged with AI grade I.

Sizing the graft for the reimplantation technique remains difficult and even though several approaches were proposed until now, there is no generally accepted approach (14, 15). The cusp height was defined as the most important determinator of the graft diameter, whereby the graft size is calculated according to a complex equation that has yet to be validated (2). On the other hand, the virtual diameter of the graft is determined by a virtual STJ diameter measured by using a prosthetic valve sizer (14, 15). In both approaches, in order to obtain a just graft size, between 5 and 8 mm are added onto the measured diameter. Suggesting that in both graft sizing methods, a subjective judgment and experience of the performing surgeon play a crucial role. Besides, both the methods assume a proper valve function, as well as morphology.

In our approach, the primary intention was to restore the vertical orientation of the IVT’s, as such the aortic root base diameter was the most important parameter to determine the graft size. The measured mean diameter in the 3D models of the AoB was 30 mm and was in average more than 60% smaller than the diameter at the STJ. The graft size was chosen based on the measured diameter, effectively providing the inner size of the AoB. Since the inner diameter of the AoB determined the graft size, *de facto* we not only downsized the STJ but also slight downsizing of the AoB was performed, this for more than 50% at the STJ and for about 5% at the level of the AoB. Within this frame, in the post-interventional echocardiography the aortic valve function was without evidence of important aortic valve insufficiency.

### 4.1. Conclusion

The AoR anatomy is complex and the key morphological elements responsible for proper valve function follow a strict well defined synchronic deformation during the cardiac cycle to ascertain proper valve function. It is more than evident that restoration of the AoR spatial architecture is a key element for successful aortic root repair with optimal aortic valve performance. In this novel approach, the 3D AoR anatomy was mapped, with focus on spatial arrangement of the morphological elements being responsible for the valve function. To our best knowledge this is a first report applying the 3D AoR morphology as a roadmap for AoR repair. The AoR reimplantation was personalized to the individual characteristics of the AoR pathology, within this a first important step toward procedure standardization was provided. Our results suggest that intraoperative sizing of the AoB is a valid method comparable to the pre-operative CT scan analysis.

### 4.2. Limitations

The study has various drawbacks allied to the low case load and retrospective nature of the data analysis. A prospective data analysis with larger cases series, including functional follow up, should be a step forward for the validation of the procedure, as well as standardization of data for a 3D model analysis. It would be mandatory in the future, beside the 3D planning, to perform in a 3D setting a functional evaluation of valve performance in short, as well as in mid period. A further approach as such is not applicable for the detailed description of the aortic root pathology in bicuspid aortic valves, warranting further additional evaluation in future.

## Data availability statement

The original contributions presented in this study are included in this article/supplementary material, further inquiries can be directed to the corresponding author.

## Ethics statement

The studies involving human participants were reviewed and approved by Ethikkomission Nordwest und Zentralschweiz, EKNZ 2021-02323. Written informed consent was not provided because, The study protocol, of this retrospective study, was approved by the local Ethical Committee at the University of Basel, Basel, Switzerland. A written informed consent specific to this study was waived due to the retrospective nature of the study. Written informed consent was not obtained from the individual(s) for the publication of any potentially identifiable images or data included in this article.

## Author contributions

GR: conceptualization, data curation, investigation, methodology, validation, and visualization. JG: conceptualization, data curation, investigation, methodology, and software. FT: data curation, supervision, and validation. EF: conceptualization, 3D data analysis, and methodology. FE: conceptualization, formal analysis, supervision, and writing—review and editing. BG: data analysis, statistical data evaluation, and writing. DB: conceptualization, data curation, formal analysis, methodology, project administration, supervision, validation, and writing—original draft. All authors contributed to the article and approved the submitted version.
